# Towards a Simulation Framework for Smart Indoor Spaces

**DOI:** 10.3390/s20247137

**Published:** 2020-12-12

**Authors:** Shadan Golestan, Ioanis Nikolaidis, Eleni Stroulia

**Affiliations:** Department of Computing Science, University of Alberta, Edmonton, AB T6G 2R3, Canada; nikolaidis@ualberta.ca (I.N.); stroulia@ualberta.ca (E.S.)

**Keywords:** smart home, sensor deployment evaluation, simulation, space model, agent model, sensor model

## Abstract

The effectiveness of sensor-based applications for smart homes and smart buildings is conditioned upon the deployment configuration of their underlying sensors. Real-world evaluation of alternative possible sensor-deployment configurations is labor-intensive, costly, and time-consuming, which implies the need for a simulation-based methodology. In this work, we report on such a methodology that supports the modeling of indoor spaces, the activities of their occupants, and the behaviors of different types of sensors. We argue that, in order for a simulation to be useful for the purpose of evaluating a sensor deployment configuration, it has to generate realistic event streams of individual sensors over time, as well as realistic compositions of sensor events within a time window. We have evaluated our simulator for smart indoor spaces, $SIM_{sis}$ toolkit, in the context of our Smart-Condo ambient-assisted living platform, supporting the observation and analysis of activities of daily living (ADLs). Our findings indicate that $SIM_{sis}$ produces realistic agent traces and sensor readings, and has the potential to support the process of developing and deploying sensor-based applications.

## 1. Introduction and Background

Research on smart indoor space (SIS) applications are receiving increasing attention from researchers and industry alike, with use cases ranging from ambient assisted living (AAL) technologies [1] to improving the efficiency and energy consumption of buildings [2]. Considering the fact that these applications have direct impact to the health and safety of their end users, i.e., the buildings’ occupants, the problem of deploying well designed configurations of sensors becomes critically important. Sensors are the entry points of smart-building applications collecting data that, in turn, are analyzed to extract useful information, which is used to guide future decisions. Today the process of sensor-deployment configuration tends to rely on the designer’s intuition and experience, or possibly on several trial-and-error experiments, an overall tedious, burdensome, time-consuming, laborious, and error-prone process [3].

Simulation tools can potentially mitigate the aforementioned problem [4,5,6]. Generally, simulators are required to realistically model indoor spaces, deployed sensors, as well as occupants. They also need to estimate the quality of a potential deployment according to potentially multiple criteria, since different smart indoor space applications have different objectives and these objectives require data to be analyzed from different perspectives. In principle, simulators should satisfy two properties: generality and external validity. Generality refers to the level of abstraction that the simulator adopts in representing the simulated scenario, which decides the range of applications that the simulator can be used for. External validity refers to how realistically and how faithfully the system can simulate a smart indoor space, the elements it contains and the activities that take place in it. General and externally valid simulators enable developers of a broad range of sensor-based applications to efficiently experiment with different sensor-deployment configurations and evaluate how effective each configuration is in collecting the raw data the application needs to meet its quality requirements.

In principle, a simulator for smart indoor spaces must model the space, the sensors embedded in it and their behaviors, and the agents that occupy the space. The *space model* defines the physical world including rooms, furniture, objects, etc. The *agent model* supports the specification of the space occupants’ and the activities they perform in the space. Finally, the *sensor model* describes the types of the deployed sensors and their sensing behavior, as well as the number and placement of the actual sensors in the space.

Space, agent, and sensor models together contribute to the overall generality and external validity of the simulator. The space model should accurately capture the geometry of the space and its interior layout. Ideally it should be general enough to describe most indoor spaces, including home, office, and corporate building layouts as well as the objects typically found in them. The agent model should accurately capture typical occupant behaviors. The model generality relies on capturing the high-level activities in which the agents are likely to engage in a given space, such as activities of daily living (ADLs) in homes, office routines in corporate buildings, etc.

Finally, the sensor model should enable the simulation of multiple types of sensors, especially those most likely to be deployed in indoor spaces. One expects from the sensor model to at least capture, with some fidelity, the fact that an agent is present within a sensor “coverage area”, or that an action was caused by the agent, e.g., flipping a switch.

We argue that the external validity of the simulator behavior should be evaluated through comparative time-series analysis of the agent-behavior and sensor-event traces it produces. In effect, given a specific scenario of a number of agents acting in an indoor space embedded with a number of sensors, the simulator should produce agent activity traces similar to the actual real-world agent activity traces and sensor-event sequences similar to the actual real-world sensor-event sequences.

More specifically, there are two important aspects in examining the validity of the simulated sensor behavior: the activation sequence of the overall set of sensors and the temporal sequence of each sensor’s readings. The former denotes the order of activated sensors in a session, and the latter denotes the values of each sensor reading throughout a session.

In this paper, we describe our work on $SIM_{sis}$, an integrated simulator for smart indoor spaces that produces sequences of realistic synthetic data sets, using space, agent, and sensor models. Our definition of space model is based on building information modeling (BIM), an industry standard for digital representation of a built facility [7], represented in the industry foundation classes (IFC) format. The IFC data model is an open specification, and an International Standard ISO 16739-1:2018, intended to describe architectural, building and construction industry data. Architectural and engineering tools use IFC to exchange data and geometry about building models between programs. This choice eliminates the need of developing special-purpose space models and enables our simulator to accommodate the complexities and idiosyncrasies of real-world buildings. Furthermore, we define a hierarchical agent-behavior model, in which virtual agents perform activities in the modeled space, towards meeting their high-level objectives. This approach enables the simulation of different occupant behaviors in different settings, such as homes or offices, where agents are likely to have different objectives and perform activities afforded by the setting. Finally, our sensor-behavior model takes into account the space geometry, the agents’ activities as well as the properties of the sensor type itself. In addition to sensors, actuators can play an important role in smart indoor space applications, but they are beyond the scope of this work.

We evaluate our simulator against a real-world case study (Smart Condo™ study [8]) and demonstrate that $SIM_{sis}$ simulations generate synthetic data similar to the data collected in the real-world deployment (our ground truth), in terms of (1) the sensor activation sequences (SAS), and (2) the temporal sensor readings (TSR).

The rest of this paper is organized as follows. Section 2 reviews several existing simulators towards their space, agent, and sensor models and investigates whether or not these models possess generalization and external-validity properties. Section 3 describes our simulation methodology with detailed explanation of each of its components, followed by a discussion of the validity metrics we propose for agent and sensor models. A simple example is provided to illustrate how the validity metrics for sensor model are applied. Section 4 details the experimental evaluation of our work and reflects on the obtained results and investigates the generalization and external-validity of our methodology, followed by the conclusion in Section 5.

## 2. Related Work

There exists a rich body of related work on simulation tools for generating synthetic data sets based on human-activity scenarios [9]. In this section, we specifically review existing SIS simulators.

Park et al. [10] proposed an early context-aware simulation system (CASS). The main purpose of their simulator is to determine whether rules triggered by sensor readings and the location of simulated users are consistent, i.e., do not result in conflicting decisions. As such, their interest is in actuation and application behavior, assuming it can be described in a rule-based fashion. Their simulator does not indicate any automated means for ingesting floorplans and/or scripting of simulated user activities unfolding over time. A configuration language takes care of all the space and sensor specification, including devices that can be actuated, e.g., air conditioner, fire alarm, dehumidifier, etc. At all times, their simulator remains a closed virtual representation of a physical environment. Conceivably, the rules, once (manually) debugged for consistency, could be transferred over to an actual system. Yet, no validation in a real environment was provided.

Buchmayr et al. [11] presented a simulator using the Microsoft .NET framework. Users are able to import a floor plan image to represent an indoor space. They simulated anonymous binary (on/off) sensors such as contact switches, motion and pressure sensors, and also temperature sensors. The simulator adds a noise signal to sensors’ actual signal and generates simulated signals for modeling faulty sensor behaviors. The simulator lacks an agent-behavior model and requires user interaction, i.e., clicking on any sensor, in order to advance its state, thus producing synthetic sensor data.

Their simulator lacks an explicit space model. In addition, the simulator’s alternative to agent model needs sophisticated and precise interactions and it is mainly subjective. Finally, the sensor model does not necessarily generalize in terms of faulty sensor behavior since the problem depends on wide range of parameters that are impossible/difficult to predict before actual implementation, hence this model cannot be validated with real-world ground truth.

Persim-3D [12], the successor of Persim [13], is a context-driven simulator in Unity3D [14]. The space model is constructed from scratch by a user through the Unity3D user interface. Their work views each sensor as belonging to one of two categories: location-based and object sensors. The former are triggered from measurements caused by the physical presence of a human agent inside their “sensing” area, e.g., a pressure sensor. The latter report a change in their state caused, directly or indirectly, by an agent, e.g., opening a door. Confusingly, they consider RFID readers as object sensors, because they report a “contact” event by reading an agent-carried RFID tag, while technically, an RFID reader also has a small coverage area. The agent model consist of actions, activities and contexts. Activities are modeled as sequence of actions and a context defines a state of simulation where a set of activities (with preconditions) are only allowed to be perform. In order to demonstrate how realistic the data produced by Persim-3D are, the authors divided real-world and synthetic data sets into subsets, in which the conditional probability of each sensor event, given the previous event is higher than a threshold. Each subset contains a sequence of sensor events up until a sensor event violates the threshold condition. Therefore, subsets are treated as different activities and sensor events are related and associated together. Then for each pair, one from synthetic data set and one from real-world data set, they evaluate if they have the same sensor events in the same order. They showed that the simulator is able to produce synthetic data 81% similar to real ones. The space model definition heavily depends on users and neglects geometrically important details and may be inaccurate. Finally, although the sensors’ behavior strongly depends on the agent’s trace, the methodology does not validate the agent model. This is important because of two reasons: first, two similar sets of sensor events could be results of two different agent behaviors, e.g., two different activities in kitchen trigger relatively the same set of motion sensors. Thus, the simulator could fail recognizing the activities, and the designer of such smart indoor spaces could use different sensor deployments to resolve the issue. Second, a specific activity, like cooking, could generate sensor events in different order. This difference can be investigated by inspecting dissimilarities in synthetic and ground-truth agent behaviors.

There are several studies (like [15,16]) based on IE Sim [17]. As with Persim-3D, a user constructs the space model within the simulator, a step that inherently endangers the accuracy of the space model. The simulator models door contact, PIR, and pressure sensors. IE Sim requires an operator to control virtual characters and perform activities by interacting with the environment.

Lundstrom et al. [15] used IE Sim to simulate ADLs. They showed that the number of PIR sensor readings over an interval follows Poisson distribution. Also Ortiz-Barrios et al. [16] statistically studied the feasibility of using IE Sim in order to generate realistic data sets. They found that since IE Sim needs a human operator, the software fails to accurately model agents in terms of activity duration. In the second study [16], the authors reported that the number of sensor events per activity is significantly similar to real-world data (with confidence level of 95% and p=0.141). If they separate the events based on the sensor type, i.e., door sensor and pressure sensor, the similarity in particular is not significant for pressure sensors. These studies examine as a validity criterion the number of sensor events per activity, and ignore the temporal ordering of these events.

Similarly, Renoux et al. [5] presented a simulator for generating ADL data sets based on their smart home application, E-care@Home. The space model should be defined by users given a floor plan. Sensors are associated to indoor objects, like a couch or oven, and their states change when a simulated human interacts with the objects, and room sensors are ambient sensors, i.e., motion and temperature sensors. Their agent model is based on a priori knowledge that provides important information about each activity, i.e., mandatory or optional, minimum and maximum duration time, earliest and latest start time, affordance objects, and prerequisites. Overall, agents organize mandatory and optional activities within a day in order to make sure mandatory activities will be performed besides as many as optional activities.

In terms of space model, indoor space definition needs a sophisticated effort in order to be accurate. In addition, there is no specific definition for indoor objects. Therefore the space model representation makes the simulation less practical and less accurate to be used for any intended application. The agent model is evaluated by asking a number of participants if each sequence of activities comes from a real or artificial agent. They found that their agent model can produce “believable” activity timeline for a session. Although, their evaluation is limited and could be subjective, their agent model mimics human behaviors accurately. However, since the agent model needs a priori knowledge, its accuracy depends mostly on expert knowledge, which could be costly in time and effort. The sensor model is also compared in terms of percentage of activation over each day, which is not adequate, because most of the smart home applications involve time-series data analysis for localization and activity recognition.

OpenSHS [18] is another smart indoor space simulator for ADL data set generation which can be used by researchers in the field of internet of things and machine learning. A designer is required to use Blender 3D [19] to design indoor spaces. Then, participants interact with the space to generate agent trace. OpenSHS supports pressure and door sensors, lock devices, appliance switches, and light controllers, and stores their readings and states according to participants interaction. The authors evaluated OpenSHS in terms of usability analysis using questionnaires given to both designers and participants, and they found the results promising. However, the synthetic data sets are required to be validated in terms of agents trances, sensors readings, and devices states. Yet again, the definition of space and agent models are burdensome and time consuming and are subject to users error.

In an interesting study [4], MASSHA, an agent-based simulator was presented for generating synthetic ADL data sets. A space model is defined by a user given a set of objects and building elements. The agent model in MASSHA is carried out by a hierarchical model where activities consist of a sequence of actions. Agents prioritize mandatory activities, and if there is no such activity, other activities are selected using a roulette-wheel approach based on their importance. They were able to model sensors in terms of frequency and duration percentage of activation during a session.

However, similar to previous attempts, the accuracy of the space model depends directly on high-effort invested by users. Objects and building elements also limit the simulator’s practical usage. The agent model enables modeling single or multi agent scenarios such as smart home or office building applications; but, it lacks validation of the model with real-world ground-truth data. If we adopt and modify the terminology used in Persim-3D to location-sensitive (LS) (for their location-based) and interaction-sensitive (IS) (for their object) sensors, what MASSHA demonstrated using their ground-truth data sets was the power of LS over IS sensors. However, the temporal granularity of MASSHA, is big for fine-grained ADL.

Masciadri et al. [6] utilized a simulator called SHARON, in order to, first, complement real-world data sets, and second, to simulate inhabitants’ activities and corresponding sensor readings. SHARON has two main layers (lacks a definition of space model): a top layer (agent model), which generates daily activity schedules based on a motivation-driven approach, and a bottom layer (sensor model), which converts the activities to corresponding sensor readings. They showed that the schedule of the generated activities is similar to real-world schedules in terms of the Earth Mover Distance metric. However, it requires real-world training sets; hence, agent model accuracy depends on having sufficient ground-truth data; thus it is not immediate, and requires real-world experiments. Additionally, the distribution of the synthetic sensor reading is compared against real-world ground truth given three specific activities. The comparison suggests that for “cleaning” and “lunch” activities, the sequences of activation were random, but for “lunch” activity the overall procedure was the same. The “shower” activity, however, had almost the same order of actions to its ground-truth peer. This comparison shows that although each activity has a set of predefined actions, they can be performed in different order, hence it is difficult to validate the behavior of simulated agents and sensors.

Based on the related work, *space models* are generally defined by a designer using an editor. In addition to efforts needed to design such space as realistically as possible, the simulator software does not necessarily offer capabilities to model any intended indoor space. Moreover, this approach makes the simulator less realistic because it may not be able to accurately take into account the complete geometry specifications.

*Agent models* are defined as virtual characters interacting with objects based on some behavioral policies, like motivation-driven [6] and hierarchy-based [4,5]. The hierarchy-based behavior can be adjusted to fit the intended context, e.g., performing ADLs, or office routines. Validation of agent models are carried out by comparing the activity distributions in simulated versus real data [6], and comparing simulator generated timeline of activities versus human generated peers [5]. The comparison by [6] is not temporal, which is necessary for smart indoor space applications. For example, we are required to anticipate the time and order of activities in order to decide about energy saving policies of a building. The comparison by Renoux et al. [5] is temporal; however, not only it could be subjective, but also it is limited to activities performed in specific time and duration. Instead, it is important to compare the temporal trace of agents based on location and activity against the real-world ground truth.

*Sensor models* are shown to be powerful when modeling LS sensors. In the research most similar to ours [4], the simulator models LS sensors for one (out of two) data sets, which has regular daily behaviors of an office space. It was found that the simulator produces hourly activation of sensors similar to ground truth. Nevertheless, this granularity level of analysis is not adequate for many smart indoor space applications.

The other sensor type that previous works mostly model are IS sensors. However, this type of sensor is heavily tailored to actions within every activity. Simulation of this sensor type can replicate real-world environments only when enough, and in the right sequence, actions are performed within every activity. For various typical indoor activities, this may be unlikely or burdensome in practice. This limitation is reported in [4,12]. More specifically, Kamara-Esteban et al. [4] found that their simulator does not produce hourly activation of sensors similar to one (out of two) of their real-world data set (single user data set), where IS sensors were deployed. The reasons were: (1) they were not able to match the real-world short-term (fine-grained) annotations in their experiments, (2) wrongly annotated activities and actions within each activity, (3) subjects not following consistent behavioral patterns during real-world experiments, and more importantly, (4) dependence of the IS sensors to sequence of actions within activities.

Table 1 summarizes the existing simulators in terms of their characteristics in terms of space, agent, and sensor models. Note that we consider the IS sensors in very broad terms, i.e., to cover even the cases where the “interaction” of the agent is indirect and may not even be uniquely and authoritatively attributed to the agent, e.g., the increase of relative humidity (RH) in a space, etc.

## 3. Simulation Methodology, Models, and Validity Metrics

**Figure 1:**
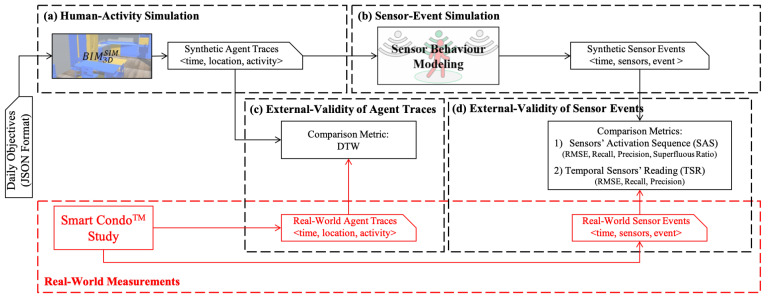
The software architecture of the $SIM_{SIS}$ toolkit. Real-world measurements module is shown in red color.

Figure 1 illustrates the architecture of the $SIM_{sis}$ toolkit. It consists of four main components: (a) human-activity simulation, (b) sensor-event simulation, (c) validity assessment of agent traces, and (d) validity assessment of sensor events. The first two components implement the simulation functionalities of the toolkit, while the latter two implement three metrics designed to evaluate the validity of the simulation and the synthetic data produced.

$BIM_{3D}^{Sim}$ [20] renders a 3D model of the simulated space based on its IFC model, extended with special-purpose object annotations specifying the user interactions that these objects afford. Virtual agents perform an activity script that meets their daily objectives, resulting in synthetic agent traces. Given these traces as input, the sensor-behavior modeling component generates synthetic sensor events. To evaluate the external validity of the synthetic agent races and corresponding sensor events, the $SIM_{sis}$ toolkit supports three metrics: dynamic time warping (DTW) for comparing synthetic agent traces against real-world agent traces, and two, sensor-sctivation sequences (SAS) and temporal sensor readings (TSR), for evaluating synthetic sensor events against corresponding real-world sensor events.

To evaluate our simulation methodology (components A and B), we compare the synthetic agent traces and sensor events it produces against a real-world study in the Smart Condo™ study [8]. In this study, participants were given a scripted sequence of typical activities of daily living, and they were asked to perform them in the order listed in their script (more details about this study is provided in Experimental Evaluation section). The Smart Condo™ space was instrumented with motion sensors and beacons, and their readings constitute the ground truth for our empirical evaluation of $SIM_{sis}$ toolkit in this paper. The ground truth regarding the participants’ movements and activities was established by manually coding video-recordings of the study, in 3-second intervals.

In principle, there are two sources of uncertainty in establishing the ground truth in such applications. First, (timestamp ambiguity), the timestamps of sensor events are inaccurate since the various data sources have different clocks which are not necessarily synchronized. Furthermore, sensor events may be lost or received by the application out of order due to problems with the network infrastructure. The second source of uncertainty (behavioral ambiguity) lies in the behavior of participants, because they might perform activities slightly (and sometimes noticeably) different. Hence, the first type of uncertainty captures impairments of the infrastructure, while the second reflects the non-uniformity or subjectivity in the execution of tasks across human populations.

### 3.1. Human-Activity Simulation

This module uses our $BIM_{3D}^{Sim}$ [20], to simulate the occupants’ activities in indoor spaces. $BIM_{3D}^{Sim}$ ingests and renders a space model using BIM files in IFC format. The BIM file (space model) includes a standard description of the indoor space layout and objects, e.g., walls, floor, furniture, etc. In addition, the space model specifies the function, e.g., bedroom, bathroom, etc., of each area in the space, where areas are modeled as convex/concave polygons that are described with a set of vertices.

$BIM_{3D}^{Sim}$ enables users to define affordances for any object in the space model; for example, a user may specify that a chair has the “sit-able” property or that a couch or a bed have the “sleep-able” property. Agents use these properties to decide on an activity that may meet one of their objectives: for example, the “resting” objective may be met by “sitting” or “sleeping”, which in turn may lead the agent to choose either the chair or the couch or the bed to “sit” or “sleep” and accomplish their objective. $BIM_{3D}^{Sim}$ receives as input the number of agents “living” in the space, their daily objectives, and the desired simulation duration *T*, and produces as output the synthetic agent traces, as a sequence of <time,location,activity> tuples.

For the purposes of this paper, an agent trace of length *N*, A is denoted as a sequence of the agent’s locations at *N* timestamps shown in Equation (1). Every two subsequent timestamps are separated by a time period τ, where τ is the frequency with which sensors emit their observations.

(1) $$\begin{array}{c} A\;=\;\left\{\left(A_{1}\right),\left(A_{2}\right),\ldots ,\left(A_{N}\right)\right\}\;=\;\left\{\left(x_{1},y_{1}\right),\left(x_{2},y_{2}\right),\ldots ,\left(x_{N},y_{N}\right)\right\} \end{array}$$

### 3.2. Sensor-Event Simulation

The sensor behavior modeling component includes a specification of the sensor configuration in the space. Each sensor is defined as a tuple s={ID,x,y,C}, where ID is the unique sensor identifier, {x,y} is the location of the sensor in the space (In the current version of the simulator, we project all locations on a 2D plane, corresponding to the floor of the space.), and *C* is the effective coverage area of the sensor.

The sensor behavior modeling component reads the synthetic agent traces as input and generates a sequence of sensor state-events (fired/unfired) for every sensor in the modeled space. In order for a sensor to fire at a particular timestamp, the agent’s location at this time has to be within the sensor’s effective coverage area. The effective coverage, *C*, can be defined as $C=C_{0}\cap G$ where $C_{0}$ is the coverage area if no geometry, e.g., occlusion and boundaries, were introduced by the space, and *G* is the part of the geometry within which the corresponding sensor is placed, i.e., boundaries imposed by the geometry of the space. Depending on where a sensor is placed, the same $C_{0}$ can result in different *C*. Moreover, depending on the particular sensor technology the geometry of the space can play a more (or less) significant role. For example, PIR sensors are limited by occlusion, while BLE beacons are much less so (their signal can pass through most residential walls and furnishings), conceptually making *G* equal to the entire 2D plane. In this paper, we conflate into *C* the impact of the unhindered coverage, $C_{0}$, and the geometry-specific impact *G*.

The synthetic sensor events capture a non-idealized sensor behavior whereby, the ${\hat{e}}_{t}^{\mathrm{ID}}=1$ and ${\hat{e}}_{t}^{\mathrm{ID}}=0$ (the synthetic sensor s.ID “fired” and “unfired”, respectively at a particular timestamp *t*) is related probabilistically to the agent’s movement to a location within the sensor’s coverage area, $C^{\mathrm{ID}}$. Specifically, we define an asymmetric error for the sensor to fire as follows:

(2) $$\begin{array}{c} Pr\left({\hat{e}}_{t}^{\mathrm{ID}}=1\;|\;A_{t}\in C^{\mathrm{ID}}\right)=\beta \left(A_{t},C^{\mathrm{ID}},{\left\{x,y\right\}}^{\mathrm{ID}}\right) \end{array}$$

(3) $$\begin{array}{c} Pr\left({\hat{e}}_{t}^{\mathrm{ID}}=0\;|\;A_{t}\in C^{\mathrm{ID}}\right)=1-\beta \left(A_{t},C^{\mathrm{ID}},{\left\{x,y\right\}}^{\mathrm{ID}}\right) \end{array}$$

where $0\leq \beta (A_{t},C^{\mathrm{ID}},{\left\{x,y\right\}}^{\mathrm{ID}})\leq 1$ captures the probability that an agent’s location $A_{t}$ at the particular timestamp *t* will be detected as such by the ID sensor, at location ${\left\{x,y\right\}}^{\mathrm{ID}}$, with an with effective coverage $C^{\mathrm{ID}}$. As an example, also used in the evaluation section and observed in several related works [21,22,23], $\beta (A_{t},C^{\mathrm{ID}},{\left\{x,y\right\}}^{\mathrm{ID}})$ can be defined as a bivariate normal distribution where the probability depends only on the distance between ${\left\{x,y\right\}}^{\mathrm{ID}}$ and $A_{t}$ when $A_{t}$ is inside $C^{\mathrm{ID}}$, and is zero otherwise (outside of $C^{\mathrm{ID}}$). The intuition of this assumption is that the closer the agent is located to the “center” of $C^{\mathrm{ID}}$, defined by ${\left\{x,y\right\}}^{\mathrm{ID}}$, the more likely it is that the sensor fires.

### 3.3. Validity of the Agent Traces

This $SIM_{sis}$ component compares ground-truth agent traces (in this paper, collected through manual annotation of videos of our real-world case study) against synthetic agent traces, using a metric based on a variant of dynamic time warping (DTW) [24] method. Based on Equation (1), we represent the real-world and the synthetic agent traces as A and $\hat{A}$, respectively. Furthermore, we adopt the Euclidean distance as the basic distance metric between two corresponding real-world and synthetic agent locations at a particular timestamp, $A_{t}$, and $\hat{A_{t}}$.

DTW temporally aligns A and $\hat{A}$ elements in order to minimize the aligning cost, producing a so-called optimal warping path ($C_{DTW}$), under certain conditions, i.e., boundary, monotonicity, and step size. An accumulated cost matrix shows the alignment cost between all the location pairs, and the optimal warping path is a path that connects pair $\left(A_{1},\hat{A_{1}}\right)$ to pair $\left(A_{n},\hat{A_{n}}\right)$, vertically, horizontally, or diagonally. Given $C_{DTW}\left(A,\;\hat{A}\right)$ as the cost of the optimal warping path found by DTW, we calculate the similarity percentage metric between A and $\hat{A}$ as follows:

(4) $$\begin{array}{c} S\left(A,\;\hat{A}\right)=\frac{C_{\mathrm{DTW}}^{\mathrm{MAX}}-C_{\mathrm{DTW}}\left(A,\;\hat{A}\right)}{C_{\mathrm{DTW}}^{\mathrm{MAX}}}\times 100 \end{array}$$

where $C_{\mathrm{DTW}}^{\mathrm{MAX}}$ is the maximum for $C_{\mathrm{DTW}}\left(A,\;\hat{A}\right)$, occurs when real-world and synthetic traces have the maximum distance between each other in every data point ($C_{\mathrm{DTW}}^{\mathrm{MAX}}=\arg\max_{i,j}\left(C_{\mathrm{DTW}}\left(A_{i},\;{\hat{A}}_{j}\right)\right)$).

### 3.4. Validity of Sensor Events

$SIM_{sis}$ compares the synthetic sensor events against their real-world counterparts using two metrics: (1) sensor activation sequence (SAS), and (2) temporal sensor readings (TSR). The former quantifies the degree to which the simulator maintains the order of fired/unfired events as compared to the real world; the latter reflects how accurately the sensor model simulates each sensor’s behavior throughout each simulation session.

#### 3.4.1. Sensor Activation Sequence (SAS)

The sensor activation sequence captures essential information about how indoor activities are being performed throughout a period of time, depending on the order of activities, and the amount of time spent on each activity. For example, in healthcare applications, caregivers can apply appropriate interventions if they observe irregularities to ADL, e.g., out of order activities, or spending too much time on a simple activity. Hence, the sequence and duration of activities need to be accurately reflected by a simulation.

Our toolkit (Figure 1) offers two metrics for evaluating the validity of the synthetic sensor events produced: the SAS metric examines whether sensor events emitted by the simulated sensors are in the same order as the events emitted by their real-world counterparts, assuming that the real-world and simulated deployment configurations are the same, including *M* sensors. We define two matrices, *E* (Equation (5)), and $\hat{E}$ (Equation (6)), as the representations of the real-world sensor events and synthetic sensor events, respectively, at *N* timestamps (columns are added with τ rate).

(5) $$E_{M\times N}=\left[\begin{array}{ccc} e_{1}^{1} & e_{2}^{1} & \cdots \\ \vdots & \ddots & \\ e_{1}^{M} & \; & e_{N}^{M} \end{array}\right],e_{n}^{m}\;\in \;\left\{0,1\right\},\;n\in \left\{1,2,\ldots ,N\right\},m\in \left\{1,2,\ldots ,M\right\}$$

(6) $$\begin{array}{c} {\hat{E}}_{M\times N}=\left[\begin{array}{ccc} {\hat{e}}_{1}^{1} & {\hat{e}}_{2}^{1} & \cdots \\ \vdots & \ddots & \\ {\hat{e}}_{1}^{M} & \; & {\hat{e}}_{N}^{M} \end{array}\right],\;{\hat{e}}_{n}^{m}\;\in \;\left\{0,1\right\},\;n\in \left\{1,2,\ldots ,N\right\},m\in \left\{1,2,\ldots ,M\right\} \end{array}$$

where every $e_{n}^{m}$ and ${\hat{e}}_{n}^{m}$ indicate the event emitted at a particular timestamp *n* by the real-world sensor with s.ID=m and its synthetic counterpart, respectively.

We compare each column in matrix *E*, $Col_{n}\left(E\right),\;n\in \left\{1,2,\ldots ,N\right\}$ against the corresponding column in matrix $\hat{E}$, $Col_{n}\left(\hat{E}\right),\;n\in \left\{1,2,\ldots ,N\right\}$ to quantify the SAS similarity, by observing the degree to which the sensor events in the two columns, i.e., elements with value greater that zero ($\left\{i\;|\;e_{n}^{i}>0\right\}$ and $\left\{i\;|\;{\hat{e}}_{n}^{i}>0\right\}$) match. We denote $Col_{n}\left(E\right)=0$ and $Col_{n}\left(\hat{E}\right)=0$ if there is no sensor event value greater than zero in column *n* in *E* and $\hat{E}$, respectively, which is the case if no sensor fired at timestamp *n*. Due to the inherent uncertainty in the timestamps of the sensor events, as discussed in Section 3, we use a windowing approach in our comparison.

Algorithm 1 describes the SAS algorithm. Given a column corresponding to timestamp *n*, for each event *q* in $Col_{n}\left(E\right)$, the algorithm determines the most similar event, $\hat{q}$, that occurred in columns of $\hat{E}$ in the window time-frame. The similarity measure in this algorithm for two events, i.e., two sensor IDs *i* and *j*, is based on Euclidean distance of sensor locations, $d_{ij}={||\left\{x,y\right\}}^{i}-{\left\{x,y\right\}}^{j}\left|\right|$, times a parameter, $\rho_{ij}$, which is the length of the shortest path between the rooms where the two sensors are located, in a graph representation of the indoor space. In the graph, every room is represented by a node, and there is an undirected edge between two nodes if and only if the rooms are adjacent. We denote θ as a special event for each column *n* if $Col_{n}\left(E\right)=0$ or $Col_{n}\left(\hat{E}\right)=0$. Accordingly, we assume the following exceptions:

(7) $$\begin{array}{c} d_{i\theta }=d_{\theta j}=d_{\mathrm{MAX}} \end{array}$$

(8) $$\begin{array}{c} \rho_{i\theta }=\rho_{\theta j}=\rho_{\mathrm{MAX}} \end{array}$$

(9) $$\begin{array}{c} d_{\theta \theta }=\rho_{\theta \theta }=0 \end{array}$$

where $d_{\mathrm{MAX}}$ is the maximum distance that two sensors could have in the space, i.e., furthest corners of the space, and $\rho_{\mathrm{MAX}}$ is the diameter of graph *G*, i.e., longest shortest path in graph *G*.

Algorithm 1 compares each reading from matrix *E* against their match in matrix $\hat{E}$ in terms of root mean squared error (RMSE), recall, precision, and superfluous ratio ($\sigma_{W}$), at different window sizes. Given a window size *W*, the superfluous ratio indicates, in average, the number of synthetic events that the algorithm did not map to any real events in each window time-frame proportion to the number of events in the window (Equation (10)). The metric is normalized, a value equal to zero shows that there is no excess synthetic events left, while any greater value shows that there were excess number of such events. The intuition behind this definition is motivated by the case where all the synthetic sensors fire all the time; in this case, the SAS algorithm would always find, for any given sensor event in the real-world matrix *E*, a matching event from matrix $\hat{E}$. Although this case results in an RMSE equal to 0 and precision and recall both equal to 1, it also exhibits the maximum superfluous ratio (≈1), implying that that synthetic sensor events in each column do not provide much information about agent traces.

(10) $$\begin{array}{c} {\bar{\sigma }}_{W}=\frac{1}{N}\sum_{n=1}^{N}\frac{|{\hat{C}}_{u}|}{|\hat{C}|} \end{array}$$

**Algorithm 1:** Finds a matching list for real-world sensor activation sequence (SAS) regarding window size and calculates metrics.
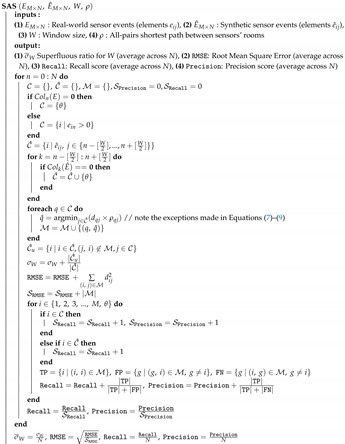


#### 3.4.2. Temporal Sensor Readings (TSR)

This metric evaluates the performance of our simulator, for each individual sensor. The ${\mathrm{ID}}_{th}$ row of matrices *E* and $\hat{E}$ represent the sequences of events emitted by the ID real-world sensor and its simulated counterpart. We compare each row from matrix *E* against its counterpart from matrix $\hat{E}$ to measure the similarity of real-world and synthetic sensor readings over time. Similarly to the SAS algorithm, we use a windowing approach to mitigate the inherent uncertainty of the phenomenon.

Algorithm 2 illustrates the process of finding a matching list for *m*-th row in matrix *E*, $R_{m}$, from *m*-th row in matrix $\hat{E}$, ${\hat{R}}_{m}$. The TSR algorithm compares each row of matrix *E* against its matching list in terms of root mean squared error (RMSE), recall, and precision, given different window sizes.
**Algorithm 2:** Finds a matching list for real-world temporal sensor readings (TSR) regarding a given window size and calculates metrics.
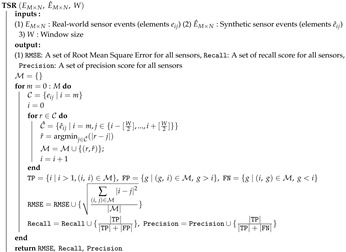


## 4. Experimental Evaluation

In order to evaluate the $SIM_{sis}$ simulator, we use the data set captured in [8] in our Smart Condo™ ∼70 m${}^{2}$ apartment. Smart Condo™ is a one-bedroom apartment unit equipped with several sensors, including motion sensors, and Bluetooth Low Energy (BLE) beacons. The condo is designated for health-related studies from different disciplines such as: medicine, rehabilitation, and computer science. In the study conducted by Mohebbi et al. [8], participants (in our paper we consider 6 of them), either alone or in pairs, performed a sequence of activities of daily living (daily objectives in Figure 1). Table 2 shows two scripted sequences of activities of daily living and their estimate time of completion; script one was given to solo participants, and paired participants each were given one of the scripts. It is worth mentioning that paired participants were asked to perform overlapped activities together.

Throughout each session, sensor data from 31 BLE beacons and 14 motion sensors are captured and stored. In addition, the location of the ground truth was produced by manual annotation of video footage from cameras in the condo. Table 3 summarizes our testbed used in this paper.

In this study, we model two off-the-shelf LS sensor types, i.e., binary infrared motion sensors and beacon sensors. The binary narrow-beam motion sensors are attached to the ceiling and fire if an agent moves within the coverage area underneath. The beacon sensors are attached to walls or objects and use the received signal strength to fire only when a transmitter is in proximity (signal stronger than −70 dBm, which translates roughly to a distance of 1 m in our setup). Similar to Mohebbi et al. [8], our simulated sensor deployment includes 14 and 31 instances of binary motion sensors and beacon sensors respectively, distributed inside of the space model with a dimension equal to Smart Condo™ (10.5 m × 6.6 m). Sensor configuration (type, ID, location, coverage area description, and room) reflects the configuration deployed in the Smart Condo™ study.

**Figure 2:**
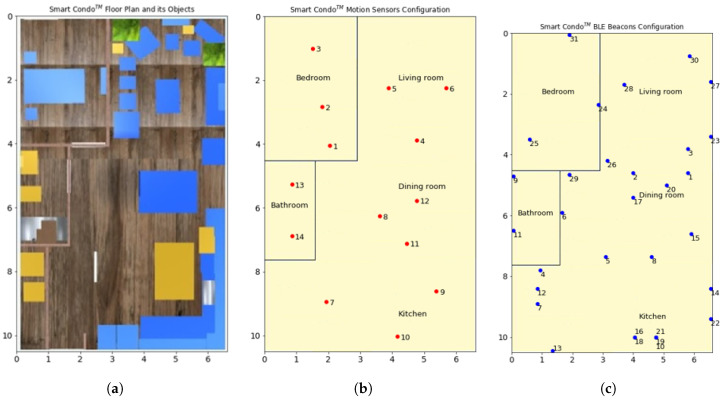
Our testbed and configuration of motion sensors and Bluetooth Low Energy (BLE) beacons. (**a**) 3D model of the Smart Condo^TM^ in $BIM_{Sim}^{3D}$ and its objects. (**b**) Motion sensor configuration in our testbed; each with an ID beside it, (**c**) BLE beacon configuration in our testbed; each with an ID beside it.

Recalling our methodology (Figure 2), we produced a 3D model of the Smart Condo™ including its objects in BIM Editor. Figure 2 shows the 3D representation of Smart Condo™ floor plan and its objects in $BIM_{3D}^{Sim}$ (Figure 2a), the location of the motion sensors (Figure 2b) and the BLE beacons (Figure 2c) in our testbed. The indoor space has five rooms, i.e., kitchen, dining room, living room, bedroom, and bathroom. The location of sensors from both types, alongside their coverage area are stored in our sensor model. We test our methodology for the four sessions shown in Table 3 to produce synthetic sensor events. We ran simulations for each session of the dataset 10 times borrowing the region of similarity idea [4], i.e., given any destination point, virtual agents can randomly select a point inside a circle with some radius around the destination. We set the radius to 1 m and report the average result.

The real-world sensor events have many outliers, i.e., false readings, that need to be removed. This is due to the fact that motion and beacon sensors are sensitive to environmental parameters such as light, noise from appliances, interference from wireless networks, etc. Therefore, we detect and remove the outliers before our evaluation.

**Outlier Removal:** We utilize real-world agents trace, A, in order to detect and remove outlier data points, from real-world sensor events. The objective was to compute a matrix, *M*, used as a mask, such that we can obtain “cleaned” sensor events by calculating Hadamard product [25] in Equation (11).

(11) $$\begin{array}{c} E_{M\times N}^{\prime }=E_{M\times N}\circ M_{M\times N} \end{array}$$

where $E^{\prime }$ is the matrix representation of the cleaned real-world sensor events. To achieve this, we calculate pairwise Euclidean distance of the real-world agents trace, A and sensors location, *S* (i.e., $S=\{{\left\{x,y\right\}}^{i}|i\in \left\{1,2,3,\ldots ,M\right\}\}$) in Equation (12), and then subtract the radius of circles circumscribing sensors’ coverage area (denoted by vector *R*) from the result in Equation (13).

(12) $$\begin{array}{c} D=|S^{T}-L| \end{array}$$

(13) $$\begin{array}{c} B=diag\left(RR^{T}\right)\;.\vec{1}-D \end{array}$$

Based on matrix *B*, we develop matrix *M* as follows:

(14) $$\begin{array}{c} M=\left\{\begin{array}{cc} 0 & \mathrm{if}B_{ij}<0\\ 1 & \mathrm{if}B_{ij}>=0 \end{array}\right. \end{array}$$

Every column in matrix *M* is a “gate” letting sensors pass their readings to the corresponding column in matrix $E^{\prime }$. However, this process is imperfect due to *uncertainty type 1*. Therefore, they are usually slightly different from each other. This phenomenon raises issues in aligning the agent traces with respective sensor events. To mitigate this issue, we use a windowing approach to obtain matrix $M^{\prime }$ as follows:

(15) $$\begin{array}{c} M^{\prime }=\left\{\begin{array}{cc} 0 & \mathrm{if}M_{i(j-\lambda :j+\epsilon )}=0\\ 1 & \mathrm{if}M_{i(j-\lambda :j+\epsilon )}=1 \end{array}\right. \end{array}$$

where λ and ϵ are our (asymmetric) window sizes (different from the one we defined for Algorithms 1 and 2) from left (prior) and right (future) sides, respectively. That is, λ is responsible to keep a history of sensor readings, whereas ϵ considers near future sensor readings. The λ and ϵ values should be assigned depending on τ. Finally the “cleaned” real-world sensor events can be represented as Equation (16). Our SAS and TSR algorithms use $E^{\prime }$ instead of *E* in our experimental evaluation.

(16) $$\begin{array}{c} E_{M\times N}^{\prime }=E_{M\times N}\circ M_{M\times N}^{\prime } \end{array}$$

### 4.1. Example

**Figure 3:**
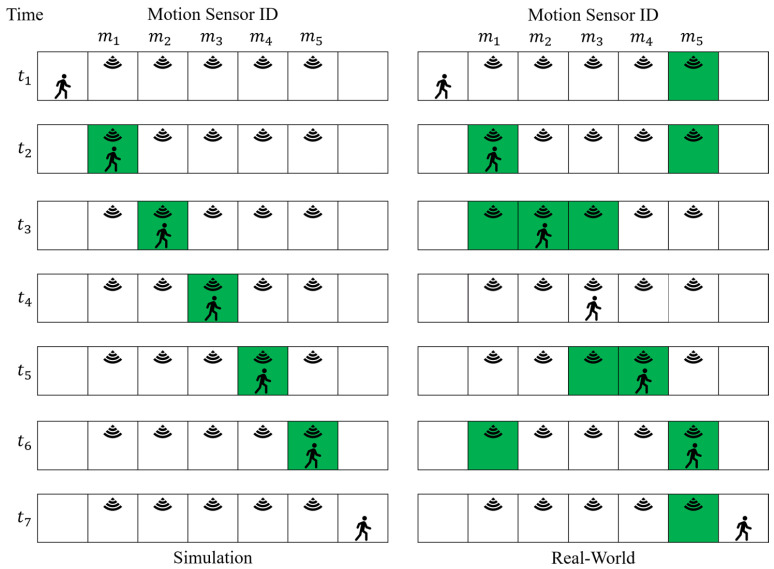
A simple environment as an example for our methodology. The green color shows which motion sensor gets activated in each step.

We demonstrate our methodology with a simple example. Consider a simple world consists of seven discrete “cells” (all the cells are part of a room) and five motion sensors (Figure 3).

An agent starts walking from the left-most cell to the right-most cell, one cell at any time unit. The coverage area of each motion sensor is the whole cell that it is located on (so the radius of circles circumscribing sensors’ coverage area is zero). The *E* and $\hat{E}$ matrices, and the vectors in Equation (1), sensors location *S*, and *R* are as follows:

(17) $$\begin{array}{c} \begin{array}{c} A=\{1,2,3,4,5,6,7\},\;S=\{2,3,4,5,6\},\;R=\{0,0,0,0,0\} \end{array} \end{array}$$

(18) $$\begin{array}{c} \begin{array}{c} E_{5\times 7}=\left[\begin{array}{ccccccc} 0 & 1 & 1 & 0 & 0 & 1 & 0\\ 0 & 0 & 1 & 0 & 0 & 0 & 0\\ 0 & 0 & 1 & 0 & 1 & 0 & 0\\ 0 & 0 & 0 & 0 & 1 & 0 & 0\\ 1 & 1 & 0 & 0 & 0 & 1 & 1 \end{array}\right] \end{array} \end{array}\begin{array}{c} \begin{array}{c} {\hat{E}}_{5\times 7}=\left[\begin{array}{ccccccc} 0 & 1 & 0 & 0 & 0 & 0 & 0\\ 0 & 0 & 1 & 0 & 0 & 0 & 0\\ 0 & 0 & 0 & 1 & 0 & 0 & 0\\ 0 & 0 & 0 & 0 & 1 & 0 & 0\\ 0 & 0 & 0 & 0 & 0 & 1 & 0 \end{array}\right] \end{array} \end{array}$$

Using Equations (11)–(16), the outlier removal filters outliers in matrix *E*:

(19) $$\begin{array}{c} \begin{array}{c} B_{5\times 7}=\left[\begin{array}{ccccccc} -1 & 0 & -1 & -2 & -3 & -4 & -5\\ -2 & -1 & 0 & -1 & -2 & -3 & -4\\ -3 & -2 & -1 & 0 & -1 & -2 & -3\\ -4 & -3 & -2 & -1 & 0 & -1 & -2\\ -5 & -4 & -3 & -2 & -1 & 0 & -1 \end{array}\right] \end{array} \end{array}\begin{array}{c} \begin{array}{c} M_{5\times 7}=\left[\begin{array}{ccccccc} 0 & 1 & 0 & 0 & 0 & 0 & 0\\ 0 & 0 & 1 & 0 & 0 & 0 & 0\\ 0 & 0 & 0 & 1 & 0 & 0 & 0\\ 0 & 0 & 0 & 0 & 1 & 0 & 0\\ 0 & 0 & 0 & 0 & 0 & 1 & 0 \end{array}\right] \end{array} \end{array}$$

(20) $$\begin{array}{c} \begin{array}{c} M_{5\times 7}^{\prime }=\left[\begin{array}{ccccccc} 1 & 1 & 1 & 0 & 0 & 0 & 0\\ 0 & 1 & 1 & 1 & 0 & 0 & 0\\ 0 & 0 & 1 & 1 & 1 & 0 & 0\\ 0 & 0 & 0 & 1 & 1 & 1 & 0\\ 0 & 0 & 0 & 0 & 1 & 1 & 1 \end{array}\right],\;\lambda =1,\;\epsilon =1 \end{array} \end{array}\begin{array}{c} \begin{array}{c} E_{5\times 7}^{\prime }=\left[\begin{array}{ccccccc} 0 & 1 & 1 & 0 & 0 & 0 & 0\\ 0 & 0 & 1 & 0 & 0 & 0 & 0\\ 0 & 0 & 1 & 0 & 1 & 0 & 0\\ 0 & 0 & 0 & 0 & 1 & 0 & 0\\ 0 & 0 & 0 & 0 & 0 & 1 & 1 \end{array}\right] \end{array} \end{array}$$

**Figure 4:**
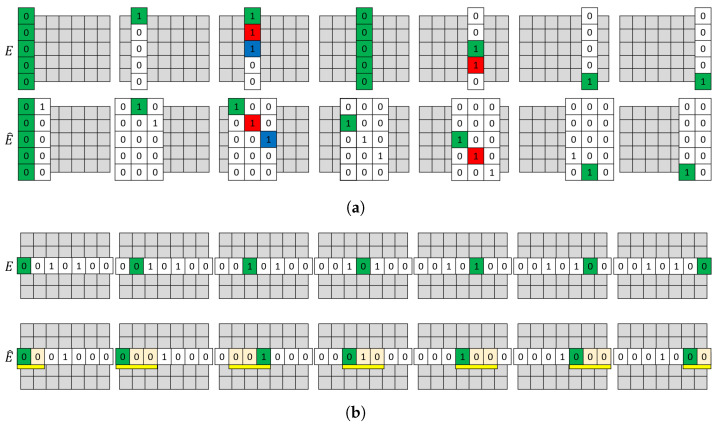
Algorithms 1 and 2 execution examples. (**a**) Algorithm 1 execution example for SAS. Colors show the algorithm’s matching. Note that $Col_{1}\left(E\right)=\theta$ and it is matched with $Col_{1}\left(\hat{E}\right)=\theta$. However, $Col_{4}\left(E\right)=\theta$ is inevitably matched with sensor *m*_2_ from $Col_{3}\left(\hat{E}\right)=\theta$ (**b**) Algorithm 2 execution example for TSR. The yellow ribbon shows the window time frame and green shows the algorithm’s matching.

Algorithms 1 and 2 compute sequence and reading matchings using columns and rows of matrices $E^{\prime }$ and $\hat{E}$, respectively. Figure 4 shows finding a matching for SAS with window size equal to 2, and finding a matching for sensor $m_{3}$’s TSR for window size equal to 2 (The λ and ϵ are both equal to 1).

### 4.2. Agent Traces Validation Results

**Figure 5:**
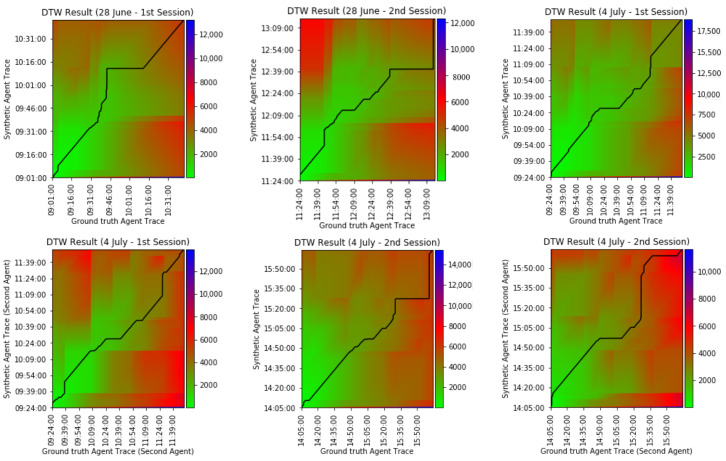
Dynamic time warping (DTW) results of synthetic agent traces compared to real-world agent traces (ground truth).

First, we compare synthetic agent traces against their real-world counterparts using the dynamic time warping (DTW) method. Figure 5 shows the DTW cost matrices for each pair of synthetic and real-world agent traces. Each element of the cost matrix, (i,j) (which is equal to $C_{DTW}\left(A_{i},\;{\hat{A}}_{j}\right)$), shows the accumulated cost of an optimal warping path starting at lower left corner, $\left(A_{1},{\hat{A}}_{1}\right)$, and ending at (i,j). Figure 5 shows the optimal warping path (black solid path) in each matrix for $(A_{N},{\hat{A}}_{N}$). Therefore, the optimal warping path shown in each matrix is actually equal to $C_{DTW}\left(A,\;\hat{A}\right)$ defined in Section 3.3. Based on the DTW algorithm, the closer the path to a diagonal line, the more similar two traces are, and therefore the higher the quality of the simulation. Table 4 shows the similarity measure ($S(A,\hat{A})$) results for our testbed. In total, our agent model is able to replicate real-world agent traces from our testbed with the accuracy of μ=82.70% (σ=13.57%). Considering the fact that in the worst and best case scenarios, virtual agent would be 12.4 m and 0 m apart from the real agent, respectively. Our results indicate that, on average, the difference between virtual agent and real agent location was 1.7 m.

### 4.3. Sensor Events Validation Results

**Figure 6:**
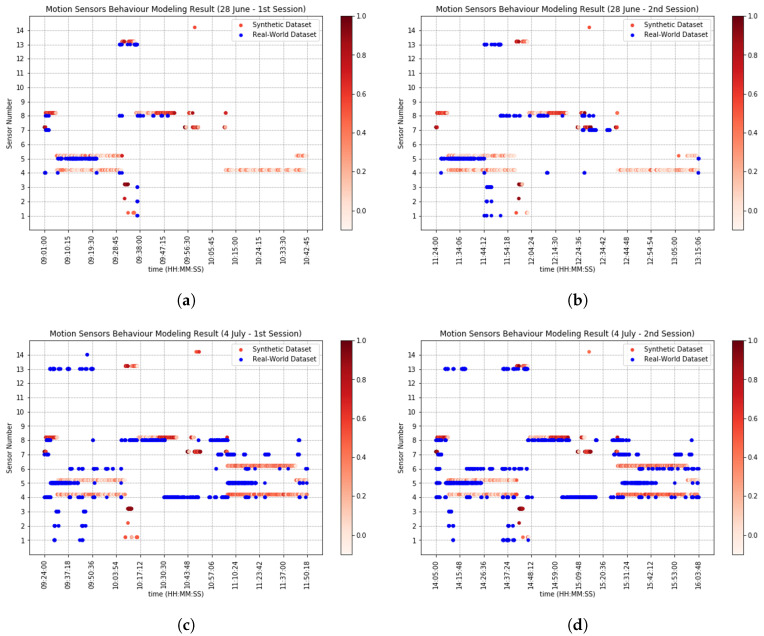
Ground-truth sensor events (blue dots) versus synthetic (red dots) for motion sensors only. Red dots are plotted slightly higher that the line of the particular sensor to avoid occlusion. (**a**) 28 June—1st session results. (**b**) 28 June—2st session results. (**c**) 4 July—1st session results. (**d**) 4 July—2nd session results.

**Figure 7:**
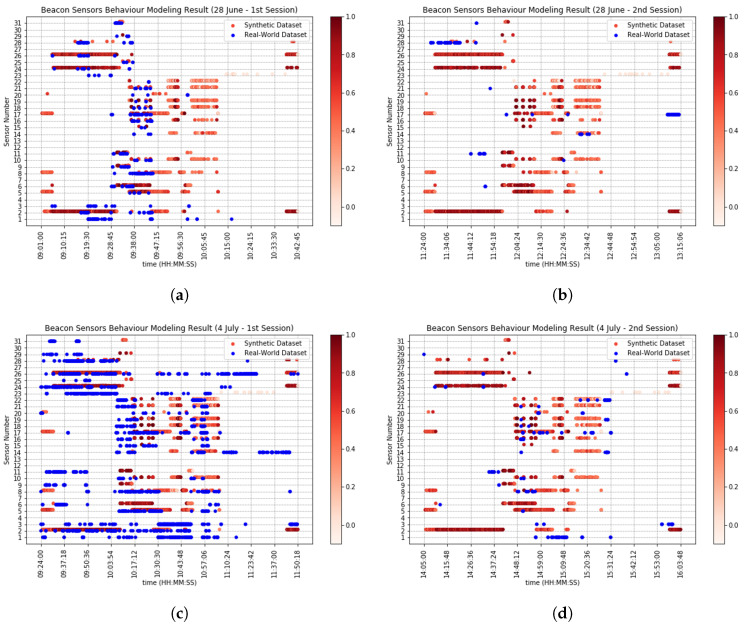
Ground-truth sensor events (blue dots) versus synthetic events (red dots) for for beacons only. Red dots are plotted slightly higher that the line of the particular sensor to avoid occlusion. (**a**) 28 June—1st session results. (**b**) 28 June—2st session results. (**c**) 4 July—1st session results. (**d**) 4 July—2nd session results.

Figure 6 and Figure 7 compare real-world and synthetic motion sensor events and beacon events, shown with blue and red dots, respectively. More red dots indicate that synthetic sensors fired more throughout the 10 times of simulation trials. Ideally, blue and red dots should align perfectly for each sensor. Nevertheless, there are differences in the synthetic sensor events and real-world sensor events in both sensor types, due to *timestamp ambiguity*, i.e., the synthetic sensors do not fire at the same time as their real-world counterparts, but also due to the agents’ *behavioral ambiguity*, i.e., synthetic agents, even when they execute the activity script of their real-world counterparts, they may do so differently.

Take as an example motion sensor 8 from Figure 6. The sensor was placed above a table where all of the kitchen appliances were placed. The sensor readings throughout four sessions are slightly different from each other, indicating that there were stochastic behaviors in real-world agents, e.g., grabbing cookware and utensils from cabinet at the same time or separately, or toasting bread while at the same time making scrambled eggs. Moreover, in the real world, motion sensors are sensitive to motions within their coverage area (which is not considered in our sensor model), which implies that if a participant walks within the coverage area underneath a motion sensor and stays still, the motion sensor only triggers when the participant was walking and does not trigger when the participant was staying still (take as an example sensor 8 from Figure 6a). Furthermore, beacons do not always send signal strength proportional to their distance with a receiver; for example, beacons could send ≥−70 dBm even when the receiver is farther than 1 m of proximity, or vice versa, the sensors could send <−70 dbm when the receiver is within 1 m of proximity.

#### 4.3.1. Sensors’ Activation Sequence (SAS) Validity

**Figure 8:**
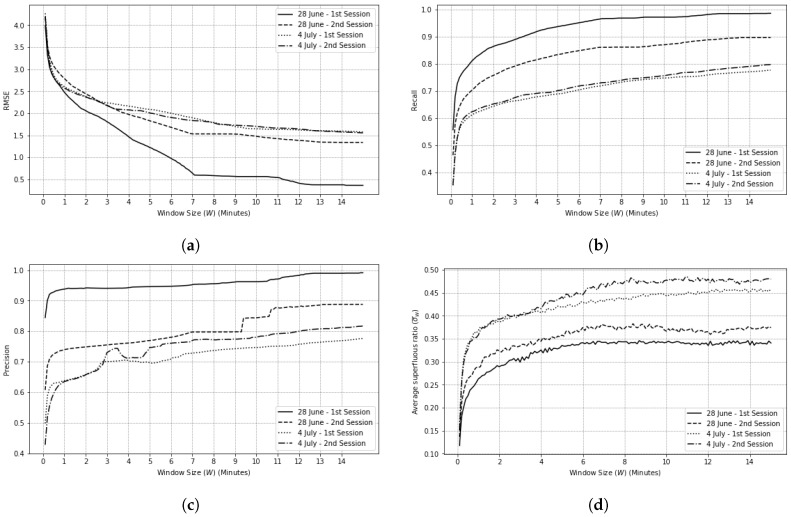
SAS analysis for motion sensors for different window sizes. (**a**) RMSE value, (**b**) recall value, (**c**) precision value, (**d**) superfluous ratio.

**Figure 9:**
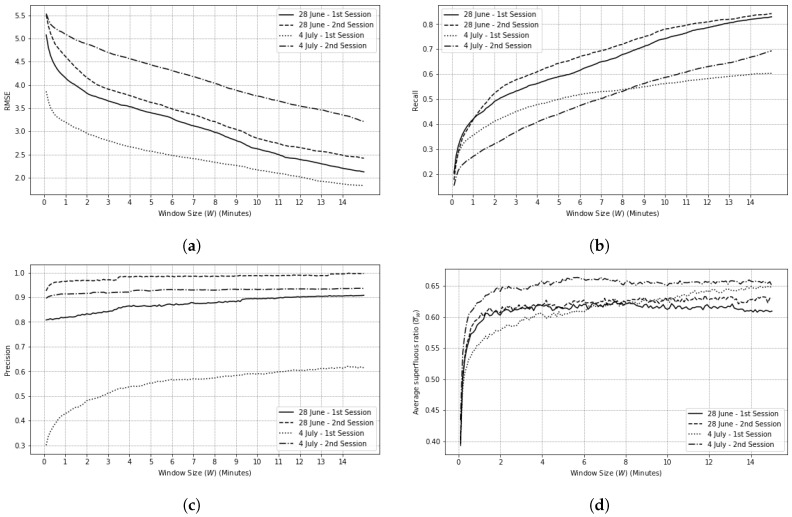
SAS analysis for beacon sensors for different window sizes. (**a**) RMSE value, (**b**) recall value, (**c**) precision value, (**d**) superfluous ratio.

For motion and beacon sensors, e∈{0,1}, and $\hat{e}\in \left\{0,1\right\}$ are elements of matrices $E^{\prime }$ and $\hat{E}$, respectively. By using these two matrices, Algorithm 1 can be executed for various window sizes. For each sensor type, the algorithm calculates a matching list M and accordingly, calculates RMSE, recall, precision, and superfluous ratio. We repeat this procedure for all of our data sets and various window sizes. The results of these metrics shown in Figure 8. In addition, we perform, separately, the same analysis for beacon sensors shown in Figure 9.

Based on Figure 8 and Figure 9, as we increase the window size, RMSE, recall, and precision improve. It is safe to say that for large enough window sizes, these metrics converge because Algorithm 1 has plenty of options to choose from in order to create a matching list for a given sensors’ activation sequence. However, larger window sizes results in higher superfluous ratio. For each window size, the precision and recall scores are calculated for each label, i.e., sensor number, and calculated their average score. Calculating precision for larger window sizes increases the chance of finding more false positives, which results in scores lower than smaller window sizes.

#### 4.3.2. Temporal Sensors’ Reading (TSR) Validity

**Figure A1:**
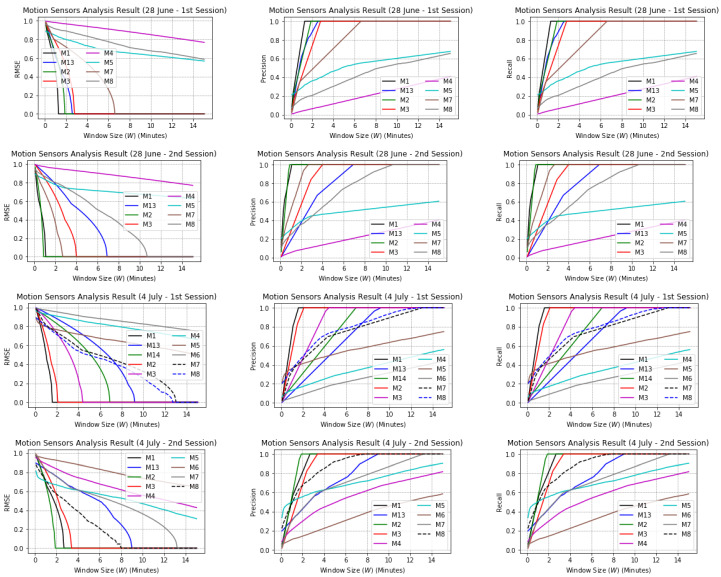
TSR analysis: (left column) RMSE value, (middle column) precision score, and (right column) recall score of motion sensors (shown in different colors) for different window sizes in each session.

**Figure A2:**
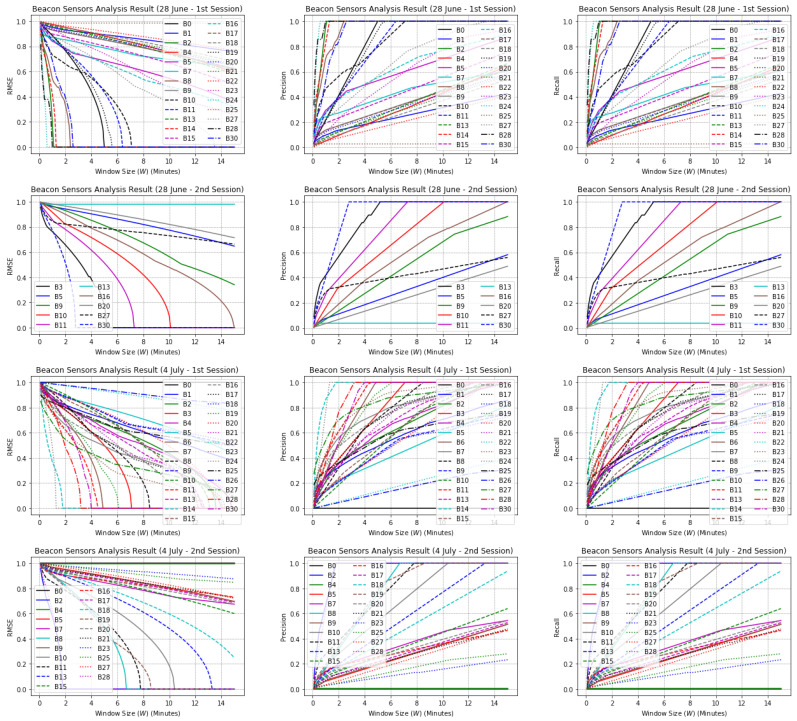
TSR analysis: (left column) RMSE value, (middle column) precision score, and (right column) recall score of beacon sensors (shown in different colors) for different window sizes in each session.

Our methodology uses Algorithm 2 with input matrices $E^{\prime }$ and $\hat{E}$ for beacon and motion sensors with different window sizes. For any calculated matching list, RMSE, precision, and recall metrics are obtained for both motion and beacon sensors, shown in Figure A1 and Figure A2, respectively, for our testbed. The figures show the better performance of sensor model for larger window sizes. An interesting observation for both motion and beacon sensors is that for large enough window sizes, sensors can be categorized into two groups in terms of their slope in RMSE, precision, and recall. The first category, i.e., the sensors with the steeper behaviors than the other category, and eventually convergence, are the sensors that our sensor model accurately simulates. Likewise, the second category are the sensors that our sensor model fails to simulate.

### 4.4. Discussion

Our methodology models space, agents, and ambient sensor behavior that produces synthetic data set similar to real-world counterparts. The space model is a BIM model, a standard format produced by several architectural tools that accurately models the geometry of any intended indoor space. Our $BIM_{3D}^{Sim}$ gets a BIM file, renders it to a 3D model. Users are able to add/define affordance properties for any object inside the model. Detailed geometry specifications of the model enables modeling agents and sensors with high fine degree of granularity.

The agent model is capable of generating synthetic agent traces based on a scripted sequence of activities, associated with daily objectives. Based on the results obtained from DTW, we observe that the model replicated real-world agent traces. In order to analyze the accuracy of the model, we obtain a “baseline model”, wherein for each entry in real-world agent traces, we generate a random location. We obtain 20 “random agent traces” for each of the participants in our testbed (120 in total). On average, the similarity measure ($S(A,\hat{A})$) between real-world agent traces and random agent traces is μ=73.62%(σ=47.68%). We assume the accuracy from both baseline model and our agent model come from normal distributions, and calculate paired t-test. By conventional criteria, the difference of the two distributions is considered to be statistically significant ($p_{value}=0.032$). However, there are flaws in interpreting the scripted activities into traces (*behavioral ambiguity*). There is a semantic gap in deciding how to perform some activities, even though following the daily objectives, e.g., one could debate how/where to perform specific activities like changing clothes, using a broom, or cooking. This usually happens to abstract activities, as compared to straightforward activities like use toilet, or take shower, which are activities unlikely to be performed differently every time, which would also cause different duration for performing those activities. That is why we see sometimes a lot of differences in Figure 5. These differences then adversely affect our sensor behavior modeling. Activities that the agent model fails to accurately model are: make tea, cooking, cleanup dining room, cleanup kitchen, iron shirts. On the other hand, activities that the agent model accurately models are: exercise, use toilet, take shower, washing hands, eat at dining table, doing laundry, watch TV, work with tablet. This analysis can be noted from Figure 5, 28 June—1st session, where at the beginning the participant performed exercise activity inside a marked area in living room. This is a straightforward activity because the participant stood on a spot and followed simple instructions shown in television. However, as it can be seen from all the figures of Figure 5, there are differences in the middle part of the figures. This is when participants were asked to cook. The cooking activity involved several actions, e.g., grab a pan, grab eggs, setup dining table, etc, which not only could introduce doubtfulness to participants, but also these activities could be performed differently every time by the participants. On the other hand, virtual agents followed the scripts.

**Figure 10:**
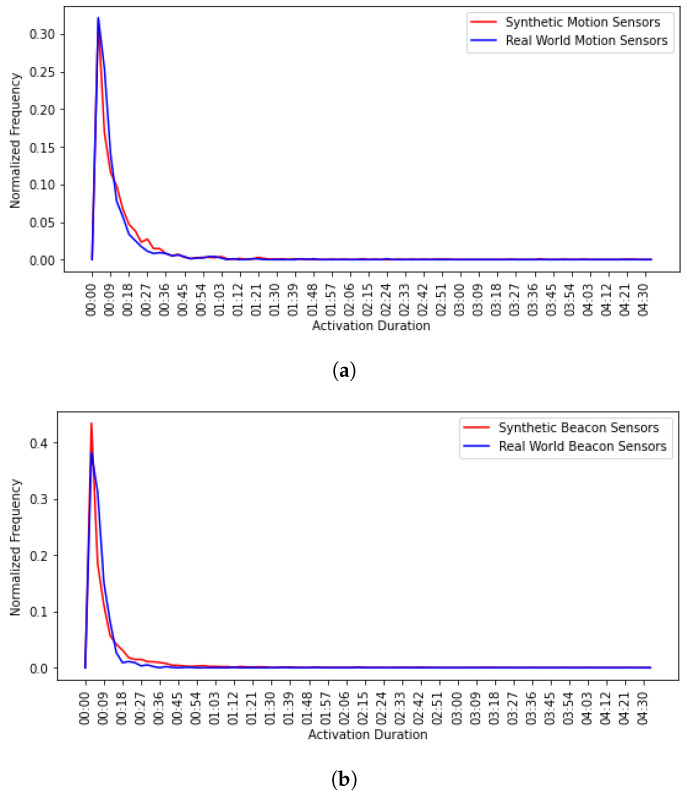
Normalized frequency of activation duration for (**a**) synthetic and real-world motion sensors, and (**b**) synthetic and real-world beacon sensors..

The sensor model replicates real-world sensors accurately for large enough (in minutes scale) timestamp granularity. This means that the time intervals in which synthetic and real-world sensors were active should have similar distributions. Figure 10 shows the normalized frequency of activation duration for both synthetic and real-world motion and beacon sensors. We can observe that the most frequent activation duration in both sensor types is 6 s; and the frequency decreases for larger activation durations. It is worth mentioning that real-world motion and beacon sensors decay faster as activation duration increases; this is because real-world sensors might not get triggered constantly for a long time.

Our sensor model generates synthetic sensor events based on the synthetic agent traces. The validity of sensor model depends on the validity of the agent model, meaning that we can only validate sensor behaviors when synthetic agent traces perfectly match real-world agent traces. An obvious example for this can be seen from Figure 5—(28 June—2nd session), at 11:44:00 from *x* axis, where the path moved vertically from 11:49:00 to 11:59:00 from *y* axis. This is due to the fact that real-world agent went to bathroom and used the toilet at 11:44:00, whereas the synthetic agent did the same at 11:59:00, so DTW algorithm warped the time in order to match the “use the toilet” activity in both traces. That is why we can see from Figure 6 that motion sensor number 13, which is placed on the top of toilet activated after some delay. We can see from Figure 5 that there are differences, sometimes huge, in synthetic and real-world traces (*behavioral ambiguity*). Based on this reason and *timestamp ambiguity*, regardless of the huge differences, and our Smart Condo™ application, we could choose window size equal to 7 min if we wish to mitigate the issues to some extent. Therefore, we report in Table 5 and Table 6 the performance of our sensor model in terms of SAS and in Table 7 the performance of the model in terms of TSR.

Table 5 shows that we had 1.44 m average error in activation sequence for motion sensors, which is small in our application. In addition, the average superfluous ratio is 0.4. However, for beacon sensors (Table 6), the error is as high as 3.18 m, with average superfluous ratio equal to 0.63. This behavior, i.e., high RMSE value and relatively low average superfluous ratio, means that beacon sensors had moderately different activation sequences. The recall score for beacon sensors also confirm this analysis. This might be due to two reasons: the noisy behavior of beacon sensors, and/or the consequence of the −70 dBm threshold to 1 m distance.

In terms of TSR, Table 7 shows that in average we had 0.31 and 0.32 sensor reading error (RMSE values for TSR) for motion and beacon sensors respectively (since they are binary sensors, the maximum is 1.0 and the minimum is 0.0 and baseline is 0.5). We should mention that the sensor model failed to model several beacon sensors due to their erratic behavior. For example, in Figure A2 (28 June—1st Session) in the Appendix, there are several beacon sensors with rapid decrease in RMSE value, but others stay above 0.2 even with large window sizes. For this reason, we remove, as outliers, sensor behaviors with RMSE >0.8 in Table 7. Precision and recall scores for both sensor types show high performance of our sensor model in modeling sensors in terms of TSR.

Our analysis shows if we assume that synthetic agent traces match real-world agent traces perfectly (not having behavioral ambiguity), then our sensor model is externally valid. However, we assumed that coverage area of sensors are circular. Although this representation is a generalized definition for ambient sensors, and IS sensors, e.g., pressure sensors, sensor model also should be capable of modeling other sensor types like temperature sensors, CO${}_{2}$ sensors, or smart objects like wearable technologies. Then the agent model could be further extended in order to generate corresponding traces to cover more smart indoor space applications, e.g., fall detection, interaction scenarios for energy consumption analysis, etc.

We intend to resolve the behavioral ambiguity in the future, i.e., fill the semantic gap in interpretation of performing activities from the perspectives of real and artificial agents. One possible solution is to simply model agents with different characteristics, e.g., movement and ability in performing activities. A more sophisticated approach is to train a generative model in order to produce realistic agent traces, while allowing re-sequencing of actions to some extent.

We also intend to simulate actuators in $SIM_{sis}$ toolkit, in order to support simulating wider range of smart indoor space applications.

## 5. Conclusions

Smart homes and buildings are a very active topic of research, with a variety of applications, from ambient-assisted living, to telecare, to occupancy analysis for energy management, relying on sensor data to provide comfort and safety to the people living and working in them. Key to the effectiveness of these applications is the proper configuration of the sensors embedded in the space, but finding a satisfactory configuration is labor-intensive, costly, and time consuming.

In this paper, we described the $SIM_{sis}$ toolkit, a simulator for indoor smart spaces, that makes the following important contributions to the state-of-the-art. First, it incorporates a high-quality model of the space, relying on BIM in the IFC format, the de-facto representation standard of building information models. IFC enables the accurate specification of the space 3D geometry and the furnishings and objects in it. $SIM_{sis}$ augments the IFC building model with specifications of the affordances of the objects in the space, so that virtual agents, given a set of objectives, move through the space and interact with the objects in it to accomplish their goals. Second, it includes a sensor-modeling-and-simulation component that realistically models sensors based on their type, location, and coverage area and simulates their event firing considering an increasing level of noise in the periphery of their coverage area.

We argue that an informative comparison between a simulation and the corresponding real-world activity should involve three dimensions of analysis.

1. Virtual agents should behave similarly to their real-world counterparts. $SIM_{sis}$ adopts dynamic time warping (DTW) as a measure of how close the sequence of the virtual-agents basic actions are to those of the real agents.
2. As the agents move and act within the space, the simulated sensors deployed in the space should behave (fire or not fire) as their real-world peers. The sensor activation sequence (SAS) metric was conceived for this purpose.
3. Finally, the sensor events emitted by a simulated sensor and its real-world counterpart over time should be similar. The temporal sensors’ reading (TSR) metric captures this type of similarity.

We have evaluated the validity of $SIM_{sis}$ simulations by comparing the synthetic traces it produced when configured with a model of the space, agent and sensors of a real-world study we conducted in the Smart Condo™. Our results demonstrate that $SIM_{sis}$ accurately simulates agents’ basic activities, i.e., moving, sitting, and standing close to objects to interact with them, but is not aware of abstract activities, i.e., cooking or sweeping the floor. The sensor-simulation component performed well in replicating the behavior of motion sensors but needs to be improved with respect to simulating beacons.

Our results demonstrate the potential of our $SIM_{sis}$ toolkit, and the simulation methodology it supports, for generating realistic agent and sensor-event traces to support the development of sensor-based applications in smart buildings. In the future, more sophisticated agent and sensor models are needed in order to cover wider range of smart indoor space applications.

## Figures and Tables

**Table 1:** Existing smart indoor space simulators.

Simulator	Space Model	Sensor Model	Agent Model
CASS [10]—2007	Floorplan Image	IS (RH, temperature, perception, and smoke sensors)	Interactive: users need to manually control an agent
Buchmayr et al. [11]—2011	Floorplan Image	LS (motion and pressure sensors) and IS (contact switches, temperature sensor)	Interactive: users need to manually control an agent
Persim-3D [12] —2015	Interactive: users need to utilize Unity 3D and an asset package	LS (pressure and vibration sensors) and IS (RFID tag, RFID receiver, and contact-detection sensors)	Model-driven: deployed a hierarchy-based approach
Lundstrom et al. [15]—2015	Interactive: users need to utilize the simulator’s UI	LS (motion, and pressure sensors) and IS (door contact)	Interactive: users need to manually control an agent
OpenSHS [18] —2017	Interactive: users need to utilize Blender 3D	LS (pressure sensor) and IS (door sensors, lock devices, appliance switches and light controllers)	Interactive: users need to manually control an agent
MASSHA. [4] —2017	Interactive: users need to utilize the simulator’s UI	LS (motion sensors) and IS (appliance switches)	Model-driven: deployed a hierarchy-based approach with randomness
Renoux et al. [5] —2018	Interactive: users are able to import a floorplan and they need to utilize the simulator’s UI	LS (motion and temperature sensors) and IS (appliance switches)	Model-driven: deployed a priori-knowledge-based approach
SHARON [6] —2018	N/A	IS (appliance switches)	Model-driven: deployed a Motivation-driven approach based on agents needs (hunger, tiredness, boredom, stress)
Ortiz-Barrios et al. [16]—2019	Interactive: users need to utilize the simulator’s UI	LS (motion, and pressure sensors) and IS (door contact)	Interactive: users need to manually control an agent

**Table 2:** Scripted sequence of activities of daily living performed by participants in [8] followed by their estimated time of completion.

Scripts	Activities		
Script 1	Exercise (30 m) Use Toilet (1 m) Change Cloths (1 m 30 s) Take Bath (2 m) Wash Hands (30 s) Fill Kettle (15 s) Make Tea (3 m 30 s) Cook Eggs (5 m 30 s)	9. Setup Table (10 s) 10. Eat Meal (7 m) 11. Take Medicine (30 s) 12. Wash and Rinse Dishes (3 m) 13. Drain Water and Wipe Cabinets (1 m)	14. Broom Kitchen (1 m) 15. Broom Dining Room (1 m 30 s) 16. Do Laundry (2 m) 17. Iron Shirt (10 m) 18. Work with Tablet (30 m) 19. Watch TV (5 m)
Script 2	Use Toilet (1 m) Change Cloths (1 m 30 s) Take Bath (2 m) Wash Hands (30 s) Work with Tablet (30 m) Fill Kettle (15 s) Make Tea (3 m 30 s) Cook Eggs (5 m 30 s)	9. Setup Table (10 s) 10. Eat Meal (7 m) 11. Take Medicine (30 s) 12. Wash and Rinse Dishes (3 m) 13. Drain Water and Wipe Cabinets (1 m)	14. Broom Kitchen (1 m) 15. Broom Dining Room (1 m 30 s) 16. Exercise (30 m) 17. Do Laundry (2 m) 18. Iron Shirt (10 m) 19. Watch TV (5 m)

**Table 3:** Our testbed.

Date	#Participants	#DataPoints	Duration	τ
28 June—1st	1	2042	01:41:00	3 (sec)
28 June—2nd	1	2229	01:51:21	3 (sec)
4 July—1st	2	2936	02:26:42	3 (sec)
4 July—2nd	2	2381	01:58:57	3 (sec)

**Table 4:** Similarity measure obtained from DTW for real-world agent traces and synthetic agent traces. The average cost of the optimal warping path.

	28 June—1st	28 June—2nd	4 July—1st (Agent 1)	4 July—1st (Agent 2)	4 July—2nd (Agent 1)	4 July—2nd (Agent 2)
$S(A,\hat{A})$	78.77%	85.61%	86.54%	86.90%	79.87%	78.56%
	Total similarity					
	μ=82.70%,σ=13.57%					

**Table 5:** SAS analysis for motion sensors with *W* = 7 min.

Motion Sensors Activation Sequence			
RMSE	Precision	Recall	Superfluous ratio
1.44 σ=0.25	0.81 σ=0.007	0.82 σ=0.009	0.40 σ=0.002

**Table 6:** SAS analysis for beacon sensors with *W* = 7 min.

Beacon Sensors Activation Sequence			
RMSE	Precision	Recall	Superfluous ratio
3.18 σ=0.38	0.83 σ=0.02	0.60 σ=0.006	0.63 σ=0.0002

**Table 7:** TSR analysis for *W* = 7 min.

*Session*	Temporal Motion Sensor Readings			Temporal Beacon Sensor Readings			
	RMSE	Precision	Recall	RMSE	Precision	Recall	Sensor Outliers Removed
28 June—1st	0.28 σ=0.14	0.77 σ=0.09	0.77 σ=0.09	0.23 σ=0.09	0.84 σ=0.04	0.84 σ=0.04	B1 B4 B9 B13 B17 B18 B20 B21 B22 B25
28 June—2nd	0.25 σ=0.11	0.81 σ=0.07	0.81 σ=0.07	0.39 σ=0.09	0.74 σ=0.05	0.74 σ=0.05	B5 B13 B20
4 July—1st	0.36 σ=0.10	0.75 σ=0.07	0.75 σ=0.07	0.35 σ=0.06	0.80 σ=0.03	0.80 σ=0.03	B0 B22 B26
4 July—2nd	0.32 σ=0.08	0.80 σ=0.04	0.80 σ=0.04	0.30 σ=0.07	0.82 σ=0.03	0.82 σ=0.03	B0 B4 B5 B7 B9 B15 B16 B17 B20 B21 B23 B25 B27
Total	0.31 σ=0.11	0.78 σ=0.07	0.78 σ=0.07	0.32 σ=0.08	0.80 σ=0.03	0.80 σ=0.03
